# Supplementary material for: Nutritional Value and Therapeutic Benefits of Dragon Fruit: A Comprehensive Review with Implications for Establishing Australian Industry Standards
Source: Molecules. 2024 Nov 30;29(23):5676. doi: 10.3390/molecules29235676 (PMC11643819; doi:10.3390/molecules29235676)
Supplement: Supplementary file 1 [file molecules-29-05676-s001.zip › molecules-3329434-supplementary.pdf]

Supplementary Materials:

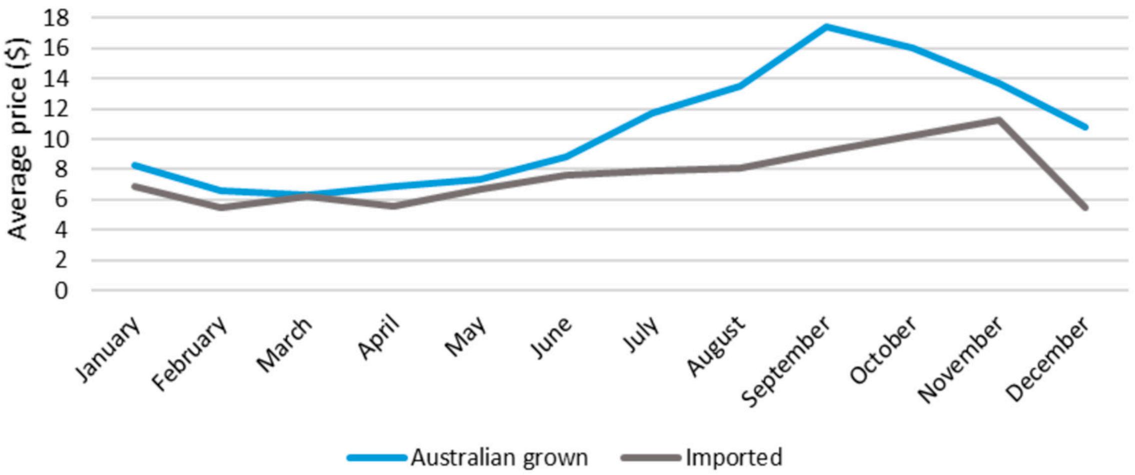

Figure S1. The monthly average price (in $ per kg) for Australian-grown and imported dragon fruit from 2014 to 2020 represents a comprehensive overview, encompassing all flesh types, markets, and packaging variations [113].

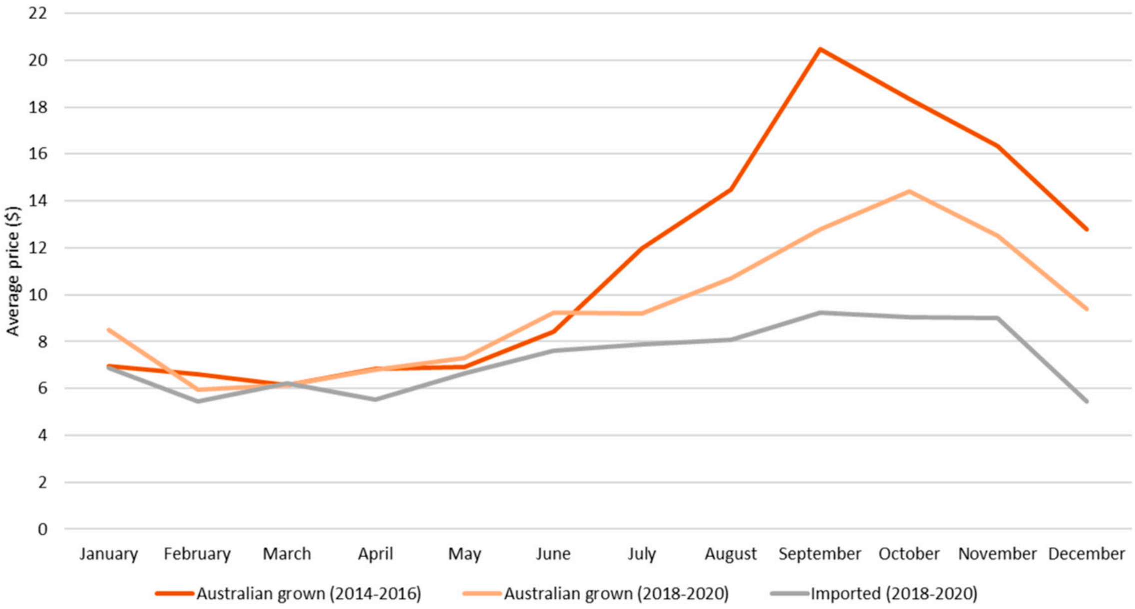

Figure S2. The monthly average price comparison for Australian-grown and imported dragon fruit, before and after the commencement of imports in 2017, includes prices across all flesh types, markets, and packaging options [113].
